# Supplementary material for: The geographical dynamics of global R&D collaboration networks in robotics: Evidence from co-patenting activities across urban areas worldwide
Source: PLoS One. 2023 Apr 13;18(4):e0281353. doi: 10.1371/journal.pone.0281353 (PMC10101514; doi:10.1371/journal.pone.0281353)
Supplement: S1 File — (DOCX) [file pone.0281353.s001.docx]

**Appendix A: Data extraction and preparation**

Table A1. Brief overview of the selected CPC symbols

| **CPC** | **Definition 3-Digit** | **Definition on most detailed level** |
| --- | --- | --- |
| B25J 9/16 | Manipulators; chambers provided with manipulation devices | Programme controls |
| B25J 9/20 |  | Fluidic |
| B25J 9/0003 |  | Home robots, i.e. small robots for domestic use |
| B25J 11/0005 |  | Manipulators having means for high-level communication with users, e.g., speech generator, face recognition means |
| B25J 11/0015 |  | Face robots, animated artificial faces for imitating human expressions |
| B60W 30 | Conjoint control of vehicle sub-units of different type or different function; control systems specially adapted for hybrid vehicles; road vehicle drive control systems for purposes not related to the control of a particular sub-unit | Purposes of road vehicle drive control systems not related to the control of a particular sub-unit, e.g., of systems using conjoint control of vehicle sub-units |
| B60W 20/30 |  | Control strategies involving selection of transmission gear ratio |
| Y10S 901 | Technical subjects covered by former USPC* cross-reference art collections [XRACS] and digests | Robots |
| G05D 1/0088 | Systems for controlling or regulating non-electric variables | characterised by the autonomous decision-making process, e.g., artificial intelligence, predefined behaviours |
| G05D 1/02 |  | Control of position or course in two dimensions |
| G05D 1/03 |  | using near-field transmission systems, e.g., inductive-loop type |
| G05D 2201/0207 |  | Unmanned vehicle for inspecting or visiting an area |
| G05D 2201/0212 |  | Driverless passenger transport vehicle |

Notes: More detailed definitions and classifications of the (sub)classes can be found at www.cooperativepatentclassification.org/cpcSchemeAndDefinitions/table; *USPC = United States Patent Classification

Figure A1. Global country R&D collaboration network in robotics

2002 – 2006


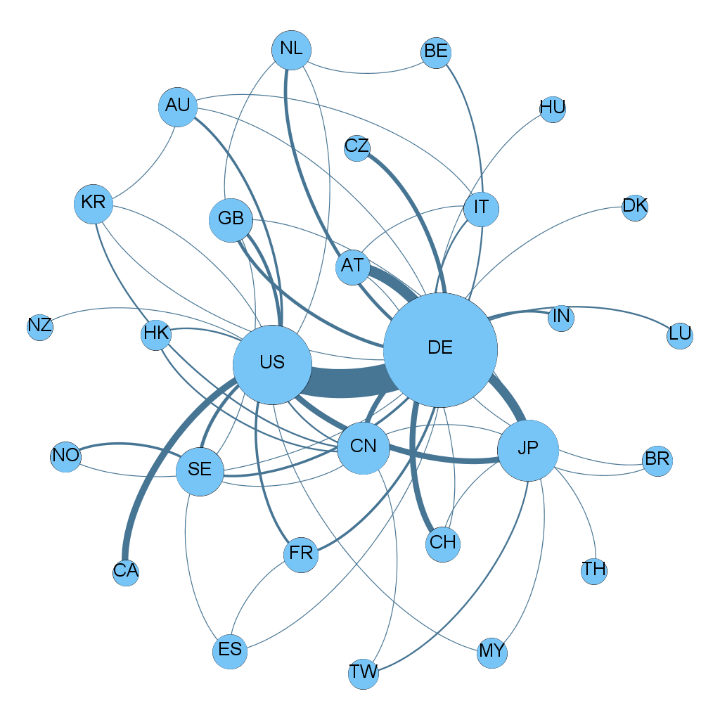


2012 – 2016


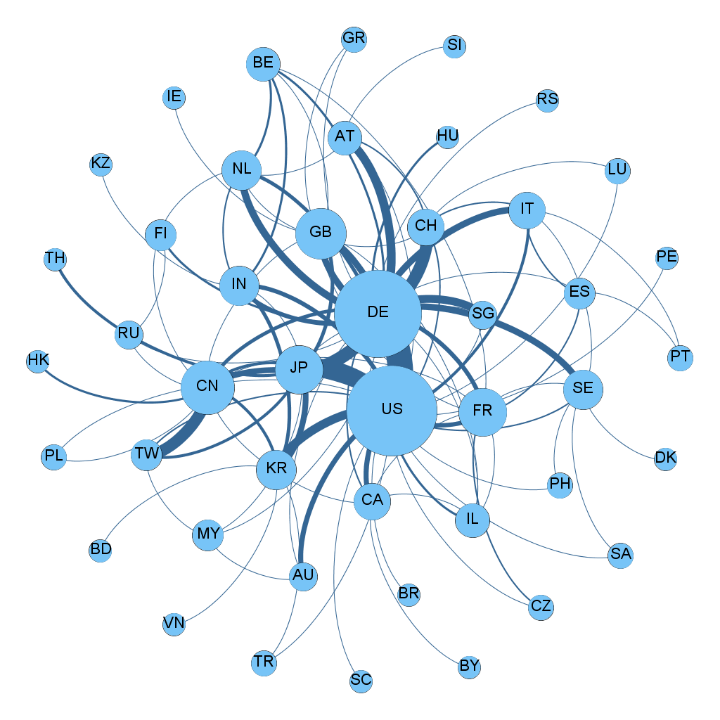


Table A2. RTA analysis of areas with total patent count greater than 2,000

| **Area** | **Patents robotics 2012-2016** | **Total patents 2012-2016** | **RTA  2012-2016** |
| --- | --- | --- | --- |
| Ann Arbor | 99.6038 | 2606.1905 | 20.0875 |
| Stockholm | 190.7605 | 5262.1416 | 19.0538 |
| Detroit | 325.539 | 9739.4012 | 17.5682 |
| Other Aichi | 399.1836 | 14594.7241 | 14.3758 |
| Kitakyushu | 88.4004 | 3480.8105 | 13.3484 |
| Stuttgart | 384.842 | 15364.3453 | 13.1651 |
| Karlsruhe | 49.3509 | 2290.7401 | 11.3233 |
| Goeteborg | 75.8621 | 3533.6226 | 11.2839 |
| Munich | 229.877 | 11622.7685 | 10.3954 |
| Frankfurt am Main | 68.307 | 4066.8358 | 8.8280 |
| Kofu | 37.2826 | 2551.9445 | 7.6787 |
| Other Michigan | 28.9795 | 2874.0367 | 5.2997 |
| Other Bayern | 47.3114 | 4912.1480 | 5.0623 |
| Other Baden-Württemberg | 47.6616 | 5334.5862 | 4.6959 |
| Regensburg | 26.0096 | 3021.1313 | 4.5250 |
| Anjo | 170.3926 | 23285.8546 | 3.8460 |
| Pittsburgh | 17.8494 | 2638.7016 | 3.5554 |
| Berlin | 29.7704 | 4476.9876 | 3.4951 |
| Changwon | 17.7179 | 2674.7968 | 3.4816 |
| Paris | 245.2848 | 37101.3071 | 3.4749 |
| Fukuoka | 31.5336 | 4860.1611 | 3.4102 |
| Nuernberg | 40.4428 | 6611.1127 | 3.2153 |
| Seattle | 82.3235 | 13620.9592 | 3.1767 |
| Dresden | 12.2429 | 2141.6889 | 3.0046 |
| Aachen | 19.3106 | 3432.7744 | 2.9567 |
| Other Nagano | 82.9888 | 15094.8380 | 2.8897 |
| Koeln | 12.8773 | 2428.8311 | 2.7867 |
| Toulouse | 20.9825 | 4251.2202 | 2.5942 |
| Heidelberg | 9.4831 | 2259.0396 | 2.2064 |
| Other Nordrhein-Westfalen | 16.0942 | 3889.4158 | 2.1749 |
| Essen | 16.3332 | 4071.0165 | 2.1087 |
| Seoul | 387.5369 | 97910.1571 | 2.0804 |
| Numazu | 18.4359 | 4661.1369 | 2.0789 |
| Mito | 22.0044 | 5903.9678 | 1.9589 |
| San Francisco | 76.87 | 22050.2013 | 1.8323 |
| San Jose | 131.719 | 38511.2902 | 1.7977 |
| Hamburg | 11.8166 | 3704.9300 | 1.6764 |
| Other South Chungcheong | 12.8802 | 4043.1245 | 1.6744 |
| Malmoe | 6.6667 | 2095.9729 | 1.6718 |
| Mannheim | 10.4022 | 3326.7849 | 1.6434 |
| Baltimore | 6.8333 | 2232.8480 | 1.6085 |
| Cleveland | 8.3664 | 2768.7451 | 1.5882 |
| Los Angeles | 39.3828 | 13746.6324 | 1.5058 |
| Boston | 54.177 | 19343.2694 | 1.4721 |
| Other Gyeonggi | 6.5666 | 2385.3474 | 1.4469 |
| Chicago | 28.083 | 10697.7723 | 1.3798 |
| Toronto | 12.6413 | 4943.5718 | 1.3440 |
| Other Yamaguchi | 18.6959 | 7946.4192 | 1.2366 |
| Hamamatsu | 46.9213 | 20090.8018 | 1.2275 |
| Raleigh | 9.8664 | 4305.2650 | 1.2045 |
| Daegu | 12.4333 | 5452.0179 | 1.1986 |
| Other North Gyeongsang | 4.5334 | 2019.1473 | 1.1801 |
| Daejeon | 34.3407 | 15695.0946 | 1.1500 |
| Mission Viejo | 4.3999 | 2059.5769 | 1.1228 |
| Grenoble | 6.6499 | 3238.4945 | 1.0793 |
| San Diego | 33.3499 | 16563.4541 | 1.0583 |
| Ulsan | 5.2856 | 2718.3200 | 1.0220 |
| Zurich | 7.2083 | 3709.4328 | 1.0214 |
| Singapore | 11.1662 | 5933.4753 | 0.9891 |
| Sendai | 8.316 | 4423.3725 | 0.9881 |
| Tel Aviv | 9.5829 | 5387.6242 | 0.9349 |
| Lyon | 4.7331 | 2696.4584 | 0.9226 |
| Helsinki | 6.5 | 3807.5031 | 0.8973 |
| Busan | 4.861 | 2848.1115 | 0.8971 |
| Duesseldorf | 4.35 | 2590.9669 | 0.8824 |
| London | 14.5452 | 8880.4578 | 0.8609 |
| Albany | 6.1836 | 3782.6148 | 0.8592 |
| Gwangju | 5.8596 | 3631.0057 | 0.8482 |
| Sapporo | 4.4997 | 2801.3830 | 0.8442 |
| Other Ontario | 5.125 | 3191.3375 | 0.8441 |
| Other South Gyeongsang | 6.3953 | 3991.3479 | 0.8422 |
| Denver | 4.6666 | 2918.1252 | 0.8405 |
| St Louis | 3.575 | 2357.5373 | 0.7970 |
| Austin | 9.7083 | 6510.5401 | 0.7838 |
| Ibaraki - rural | 26.8384 | 19138.7834 | 0.7371 |
| Utsunomiya | 5.9583 | 4806.6155 | 0.6515 |
| Other Hokkaidō | 6.2046 | 5233.5838 | 0.6231 |
| Miami | 3.3334 | 2858.1512 | 0.6130 |
| Taipei | 32.6736 | 28195.9127 | 0.6091 |
| Copenhagen | 3.3498 | 3208.5792 | 0.5487 |
| Other Shizuoka | 12.9407 | 12900.2024 | 0.5272 |
| Toyohashi | 2.3667 | 2459.5703 | 0.5058 |
| Chengdu | 11.8917 | 12366.6973 | 0.5054 |
| Washington DC | 4.712 | 4924.8535 | 0.5029 |
| Jiaxing | 2.2025 | 2306.0182 | 0.5020 |
| Concord | 3.7736 | 3980.5257 | 0.4983 |
| Kaohsiung | 11.2617 | 12018.4671 | 0.4925 |
| Minneapolis | 8.2997 | 8912.4842 | 0.4895 |
| Maebashi | 3.6666 | 4242.1182 | 0.4543 |
| Atlanta | 4.6668 | 5400.6588 | 0.4542 |
| Zhenjiang | 2.2 | 2677.9124 | 0.4318 |
| Phoenix | 3.4333 | 4204.2168 | 0.4292 |
| Dallas | 5.1168 | 6459.8771 | 0.4163 |
| Tokyo | 552.7322 | 727655.5067 | 0.3992 |
| Portland | 4.3332 | 6126.6939 | 0.3717 |
| Rochester | 2.7499 | 3948.5149 | 0.3660 |
| Nagano | 1.6999 | 2497.5236 | 0.3577 |
| Other Tochigi | 2.65 | 4151.4386 | 0.3355 |
| Other Zhejiang | 1.5 | 2396.1296 | 0.3290 |
| Eindhoven | 3.7916 | 6239.5121 | 0.3194 |
| Hiroshima | 2.95 | 4914.7498 | 0.3155 |
| Hartford | 2.0595 | 3493.8062 | 0.3098 |
| Shanghai | 65.9938 | 112058.7055 | 0.3095 |
| Osaka | 155.268 | 264757.9038 | 0.3082 |
| Toyama | 2 | 3543.1130 | 0.2967 |
| Other Niigata | 1.3333 | 2433.7469 | 0.2879 |
| Matsuyama | 2.6666 | 5068.3380 | 0.2765 |
| Basel | 2.3333 | 4446.9228 | 0.2758 |
| Himeji | 1.5833 | 3025.9206 | 0.2750 |
| Milan | 1.3334 | 2673.9432 | 0.2621 |
| Zhuzhou | 1.1429 | 2436.9521 | 0.2465 |
| Delhi | 1 | 2156.5920 | 0.2437 |
| Nagoya | 40.4394 | 87802.9153 | 0.2421 |
| Changsha | 3.0001 | 6519.8573 | 0.2419 |
| Indianapolis | 1.1166 | 2583.7355 | 0.2271 |
| Cincinnati | 1.3 | 3120.2653 | 0.2190 |
| Houston | 4.075 | 9858.3069 | 0.2173 |
| Chongqing | 2 | 4906.5910 | 0.2142 |
| Orlando | 0.9999 | 2460.0726 | 0.2136 |
| Qingdao | 6.1028 | 15332.5896 | 0.2092 |
| Changchun | 1.475 | 3879.1760 | 0.1999 |
| Gangwon | 1.0834 | 2873.4009 | 0.1982 |
| Other Jiangxi | 3.1039 | 8301.0692 | 0.1965 |
| Montreal | 0.9583 | 2676.7851 | 0.1882 |
| Datong | 0.8191 | 2440.7227 | 0.1764 |
| Guangzhou | 92.6418 | 280784.8325 | 0.1734 |
| Philadelphia | 2.2 | 6793.5031 | 0.1702 |
| New York City | 12.6164 | 39354.0552 | 0.1685 |
| Shenyang | 1.4585 | 4731.5508 | 0.1620 |
| Other Guangxi | 1.1 | 3653.8960 | 0.1582 |
| Other New York | 1.4833 | 5024.4832 | 0.1552 |
| Wuhan | 4.2833 | 15104.5888 | 0.1490 |
| Beijing | 46.1814 | 190472.4488 | 0.1274 |
| Hong Kong | 0.9916 | 4143.0957 | 0.1258 |
| Ningbo | 0.9 | 3893.5575 | 0.1215 |
| Yokkaichi | 1.1191 | 5122.7893 | 0.1148 |
| Niigata | 0.6222 | 2865.3574 | 0.1141 |
| Hefei | 0.75 | 3461.5485 | 0.1139 |
| Other Jiangsu | 2.2193 | 10411.0572 | 0.1120 |
| Bengaluru | 1.35 | 6501.0012 | 0.1091 |
| Nanjing | 3.2859 | 16427.0728 | 0.1051 |
| Tianjin | 2.2946 | 12667.9345 | 0.0952 |
| Hangzhou | 2.5262 | 14491.0786 | 0.0916 |
| Dalian | 0.604 | 3469.1682 | 0.0915 |
| Nantong | 1.05 | 6488.0747 | 0.0851 |
| Other Sichuan | 0.3429 | 2158.4521 | 0.0835 |
| Other Fujian | 1.575 | 10086.0397 | 0.0821 |
| Fuzhou | 0.3333 | 2361.5136 | 0.0742 |
| Other Hubei | 0.4167 | 2989.2101 | 0.0733 |
| Weifang | 0.25 | 2093.6808 | 0.0628 |
| Sydney | 0.3333 | 2802.0638 | 0.0625 |
| Other Liaoning | 0.2679 | 2266.3547 | 0.0621 |
| Vancouver | 0.25 | 2259.7940 | 0.0581 |
| Shantou | 0.3052 | 2957.0524 | 0.0542 |
| Jilin | 0.25 | 2424.5140 | 0.0542 |
| Jinan | 0.625 | 6255.1375 | 0.0525 |
| Zhuhai | 0.5929 | 6122.1204 | 0.0509 |
| Other Guangdong | 0.4 | 4158.3924 | 0.0506 |
| Other Hebei | 0.2917 | 3383.2289 | 0.0453 |
| Moscow | 0.45 | 6465.4635 | 0.0366 |

Table A3. Top three actors in top ten RTA areas (2012-2016)

| **Area** | **Patent applicant** |
| --- | --- |
| Ann Arbor | Ford Global Technologies, LLC |
|  | GM Global Technology Operations LLC |
|  | Toyota Motor Engineering & Manufacturing North America, Inc |
| Stockholm | Aktiebolaget Electrolux |
|  | Husqvarna AB |
|  | Scania CV AB |
| Detroit | Ford Global Technologies, LLC |
|  | GM Global Technology Operations LLC |
|  | Nissan North America, Inc. |
| Other Aichi | AISIN AW CO LTD |
|  | Toyota Jidosha Kabushiki Kaisha |
|  | TOYOTA MOTOR CORP |
| Kitakyushu | Kabushiki Kaisha Yaskawa Denki |
|  | National Institute of Advanced Industrial Science and Technology |
|  | YASKAWA ELECTRIC CORP |
| Stuttgart | DAIMLER AG |
|  | Robert Bosch GmbH |
|  | Valeo Schalter und Sensoren GmbH |
| Karlsruhe | DAIMLER AG |
|  | Robert Bosch GmbH |
|  | SEW-EURODRIVE GMBH & CO KG |
| Goeteborg | Volvo Car Corporation |
|  | VOLVO TRUCK CORPORATION |
| Munich | Bayerische Motoren Werke Aktiengesellschaft |
|  | Kuka Roboter GmbH |
|  | MAN Truck & Bus AG |
| Frankfurt am Main | Continental Teves AG & Co. OHG |
|  | GM GLOBAL TECHNOLOGY OPERATIONS LLC |
|  | Honda Research Institute Europe GmbH |
