# Supplementary material for: The geographical dynamics of global R&D collaboration networks in robotics: Evidence from co-patenting activities across urban areas worldwide
Source: PLoS One. 2023 Apr 13;18(4):e0281353. doi: 10.1371/journal.pone.0281353 (PMC10101514; doi:10.1371/journal.pone.0281353)
Supplement: Attachment — Submitted filename: Paper_review.docx [file pone.0281353.s002.docx]

Thank you for giving me the opportunity to read this study, in which you propose an interesting empirical case on the geographical localization of robotic patents. I think that your paper makes a timely and relevant contribution to make sense of the spatial emergence of this new industry that is already having profound impacts on the economy. However, I noticed a number of drawbacks to address, of which I believe the most urgent to tackle is to better ground your contribution onto the existing literature on the geography of innovation. Good luck with the revision!

**1- Introduction**

1.1-The growth of the robotics industry is presented as an opportunity for economic growth and competitiveness. However, there is no acknowledgement of the discussion about the societal challenges implied by it, particularly in terms of job losses (Frey and Osborne, 2017; Acemoglu and Restrepo, 2020), but more widely in the whole re-organization of societal functions and their impact in urban life (Macrorie et al., 2019). I understand that these discussions are not your key concern; however, you say that robots could address important societal challenges, but at the same time you might acknowledge that they could generate other problems.

1.2- The objectives of the study are somewhat general and not very connected to a gap in the literature. You say at page 3 that: “few studies exist that systematically trace the global landscape of robotic innovations and the related global R&D collaboration networks behind it”. Yet this does not explain why we need more studies on this, and what you would add to the existing literature. The fact that robotics are a very innovative technology is not sufficient, as other technologies could also be interesting to study including bio-engineering, artificial intelligence, internet of things or others. Also, many studies have geocoded patents, so I think you should play down the extent to which this is new, or explain how it differs from other contributions in this respect (see for example: Morrison et al., 2017; de Rassenfosse et al., 2019; Miguelez et al., 2019).

1.3- Audretsch and Feldmann, 1996: Reference is missing in bibliography

**2- Literature review**

2.1- Related to the previous remark, the paper should be better embedded in the relevant literature. In fact, you discuss mainly applied contributions to the topic of robotics, but your motivation for the study is to understand the geographical dimension of R&D activities in this sector. You mention collaboration networks and knowledge exchange dynamics, and there is a huge literature on economic geography and the geography of innovation of which you could at least mention some of the main traits, authors and debates. I can’t resume here this literature but it includes discussions on the extent to which different forms of proximity favor networking, on knowledge complexity, on regional branching and relatedness and many other topics (you might check Balland and Boschma, 2021 for a recent application of some of these concepts on Industry 4.0 technologies, including robotics). These approaches are likely not all necessary for your point, but I believe you should ground your contribution onto some conceptualization of R&D and innovation dynamics in space, that is now lacking, and the related debates to which your paper contributes.

**3-Methods**

3.1- Perhaps you could explain better the definition of urban areas that is used, or refer to an established definition (OECD?).

3.2- In the RTA formula, the right part at the denominator you sum over *i* twice, whereas you should sum over *i* and *k*. Also, it might be clearer if you put it graphically this way:

(From Boschma et al., 2015).

3.3- Page 8 last paragraph, the following phrase has some syntactic problem (missing verbs?):

In general, SNA has come into fairly wide use for the analysis of social systems, been to be interpreted at the individual level of socially interacting individuals

3.4- At page 9, first paragraph, adjacency matrix as defined by equation (~~1~~ 2)

3.5- At pages 9 and 10, the lists of network measures is a bit too long and probably not necessary as they are fairly well known. You could refer to Wasserman and Faust for a full definition and just list the measures used and what they show in general, using half the space.

**4- Results**

4.1- Table 1 is unclear. In fact, while the title is “RTA analysis”, the first three fields are not RTA indexes, because RTA appears only in the last field. This raises two questions: if the first three fields represent patent counts, why are they not integer numbers? If you have used fractional counts, assigning a proportional contribution to inventors in different cities, you should mention it before, so the reader can interpret these results. Second question: how can you say anything about the evolution of specialization if you only show RTA for the period 2012-2016 and not the previous one? The increase in absolute patent numbers could be simply an indication of the aggregate growth of patenting activity.

4.2- Related to this, I have some doubts that these results can support the findings you report at page 11. Particularly, in finding number 2 you say that two areas have shifted their focus to robotic R&D, but even though these numbers could suggest it, they are not enough to state it confidently. These data only say part of the story, so that for example, this might be part of a common growth in robotic specialization, or other technology fields in these urban areas might be growing even more than robotics. Hence, I suggest to nuance a bit more these statements.

4.3- Page 12, first paragraph: you say that “most urban areas show a strong specialization in the automotive industry”, but what you want to say is that applicants in these regions are mostly automotive companies. Your data do not allow you to say much about the overall specialization of these areas.

4.4-Same page-same paragraph as above, why would you expect universities or research organization to be at the top of the list? Also, data about higher share of research facilities in the Asian cities you mention is not disclosed.

4.5- The table A5 you mention at page 11 does not exist in the appendix. Is it A3?

4.6- Figure 1 and the related commentaries suggest that there has been a general increase more than decrease of specialization in robotics (related to commentary 4.2). This should be acknowledged somewhere.

**5-Network results**

5.1- In figure 2, in the notes it is not clear to what “number” link size refers to.

5.2- There doesn’t appear to be any table A8 or figure 5.

5.3- Page 17, third paragraph: “places on earth” sounds vague.

5.4- In this section you use three different measures of centrality. To which in particular do you refer when you talk of “network centrality” (p.15)? Do you consider them as being equally important or you give priority to one over others? What is the value-added of considering different measures of centrality?

**6- Discussion**

6.1- Page 18 last paragraph, you say that robotics “has grown immense”. However, you should contextualize this finding, because we know that patenting in general has greatly increased in past decades. At the same time, growth in robotics could be relativized against growth in other emergent sectors. Otherwise, this claim does not appear very substantiated.

6.2 –I do not see how the limited evidence presented in table A3 can support such vast claim as the fourth one. The remark is interesting, but you should support it with further evidence, perhaps including a larger number of patent applicants and extending research beyond the top 10 RTA areas.

6.3- Data in support of the fifth claim is not presented (see comment 4.4).

6.4- The discussion does not explain how research findings relate to existing debates and research directions in the literature (see also comments 1.2 and 2.1).

**References**

Acemoglu D, Restrepo P. Robots and Jobs: Evidence from US Labor Markets. journal of political economy. : 57.

Balland P-A, Boschma R. Mapping the potentials of regions in Europe to contribute to new knowledge production in Industry 4.0 technologies. Regional Studies. 2021;55: 1652–1666. doi:[10.1080/00343404.2021.1900557](https://doi.org/10.1080/00343404.2021.1900557)

Boschma R, Balland P-A, Kogler DF. Relatedness and technological change in cities: the rise and fall of technological knowledge in US metropolitan areas from 1981 to 2010. Industrial and Corporate Change. 2015;24: 223–250. doi:[10.1093/icc/dtu012](https://doi.org/10.1093/icc/dtu012)

de Rassenfosse G, Kozak J, Seliger F. Geocoding of worldwide patent data. Sci Data. 2019;6: 260. doi:[10.1038/s41597-019-0264-6](https://doi.org/10.1038/s41597-019-0264-6)

Frey CB, Osborne MA. The future of employment: How susceptible are jobs to computerisation? Technological Forecasting and Social Change. 2017;114: 254–280. doi:[10.1016/j.techfore.2016.08.019](https://doi.org/10.1016/j.techfore.2016.08.019)

Macrorie R, Marvin S, While A. Robotics and automation in the city: a research agenda. Urban Geography. 2021;42: 197–217. doi:[10.1080/02723638.2019.1698868](https://doi.org/10.1080/02723638.2019.1698868)

Miguelez E, Raffo J, Chacua C, Coda-Zabetta M, Yin D, Lissoni F, et al. Tied in: the Global Network of Local Innovation. : 55.

Morrison G, Riccaboni M, Pammolli F. Disambiguation of patent inventors and assignees using high-resolution geolocation data. Sci Data. 2017;4: 170064. doi:[10.1038/sdata.2017.64](https://doi.org/10.1038/sdata.2017.64)
