# Supplementary material for: The geographical dynamics of global R&D collaboration networks in robotics: Evidence from co-patenting activities across urban areas worldwide
Source: PLoS One. 2023 Apr 13;18(4):e0281353. doi: 10.1371/journal.pone.0281353 (PMC10101514; doi:10.1371/journal.pone.0281353)
Supplement: Attachment — Submitted filename: response_final.docx [file pone.0281353.s003.docx]

**Response to reviewer comments (Manuscript ID PONE-D-21-39533)**

We thank the referees for their highly valuable comments and very useful suggestions on the first version of our manuscript. We have carefully considered the remarks and have taken them into account in a revised version of our manuscript, mainly in terms of a

- more comprehensive embedding of the study in related theoretical debates of economic geography,
- a better motivation of the focus on robotics, and
- a more precise presentation of the empirical setting and respective findings (methods and data used, spatial configuration, interpretation of empirical results).

In what follows, we describe in some detail how we dealt with each of the remarks in the revised version of the manuscript.

**Reviewer #1**

1. ***What is the reference to choose the particular CPCs of robotics?***

We thank the referee for this question, clearly pointing to a weak and insufficient explanation in the original manuscript. We have revised the manuscript extending the description on how we have defined and empirically delimited robotics patents (see Section 3 of the revised manuscript, and Appendix A). Our answer is as follows:

i) In order to identify R&D activities and networks in robotics, we make use of patents and of cross-area co-patents (having at least two inventors from two different spatial areas) following earlier studies investigating international R&D collaboration networks (see, e.g. Hoekman et al. 2009, Hu et al. 2013, Lata et al. 2015).
ii) Our sample is comprised of 18,184 robotic-patents in the years 2002-2016 (extracted from the RISIS patent database) with a total of 47,281 inventor locations. In the delimitation of robotics patents, we follow a tailor made patent mapping developed by Keisner et al. (2015) who adapted a search strategy from the seminal work by UKIPO (2014).
iii) In this study, we exactly use the 13 CPC symbols as suggested by Keisner et al (2015). CPC is an abbreviation of “Cooperative Patent Classification”, which is a joint classification system between the European Patent Office (EPO) and the United States Patent and Trademark Office (USPTO), and an extension of the International Patent Classification (IPC) system administered by the World Intellectual Property Organization (WIPO). CPC is an internationally recognized patent classification system and hence a fitting reference point for classifications and searching patent applications in robotics. Its unified system is organized in a hierarchical way. More precisely, the technology fields are separated into nine sections (A-H and Y) with approximately 250.000 classification entries.
iv) The respective 13 CPC symbols are provided in Table A1 of the Appendix, accompanied with a detailed textual description.
v) The choice of the 13 CPC symbols put forward by the robotics study of Keisner et al. (2015) were subject to several robustness checks, for instance by benchmarking against lists of seminal patents and a list of patents of robotics companies.
vi) We have made these issues more clear in the methodology section of the revised manuscript and adding some more references.
2. ***What is the reference of choose CPCs of robotics? Although robotics is a knowledge intensive industry, the basic technologies also matter. What is the role of the fundamental technologies?***

This comment is quite similar as the previous one, extending a bit the issue in terms of the role of fundamental technologies. We clarified this point in the revised manuscript, underlining the scope of the study on technological, not basic or fundamental knowledge production.

i) As described in some more detail in the response to the first comment, the reference of using CPC classes for delimitating robotics related patents is Keisner et al (2015).
ii) We fully agree with the referee that basic knowledge creation and R&D, in particular in the ICT sector, are of high relevance for technological development as a whole, and clearly also in robotics.
iii) However, we stick the scope of this study clearly to technological knowledge production, i.e. the production of new knowledge focusing on inventions with the potential for generating innovation in form of new products and/or production processes.
iv) The focus on technological R&D and related technological R&D collaboration comes by definition with the mobilization of patents and co-patents as marker for R&D hot spots and networks, respectively.
v) Looking at fundamental research as well would require indicators capturing basic research activities (mainly publications, but partly also R&D projects; see Scherngell 2021 for an overview), and also developing a harmonized technological identification strategy across these indicators, i.e. an ontology for capturing robotics relevant fundamental research which goes well beyond of the focus of the study at hand.
vi) However, we have taken the argument and mentioned under future research directions this very important aspects mentioned by the referee.
3. ***How to define the concept of urban area? What is the delineation? And What is the standard to choose the 900 urban areas?***

We have looked at the original manuscript and fully agree that there are some unclarities and inconsistencies in the description of the spatial configuration. We have substantially clarified this issue in the methodology section of the revised manuscript (see Section 3). The main points in this context are the following:

i) We know very well from the literature in human geography, that socio-economic phenomena are usually unevenly distributed within countries (referred to as spatial disparities or inequalities), and here in particular innovation and R&D activities (see Audretsch and Feldmann 1994).
ii) Therefore, given the focus of this study aiming to investigate global R&D collaboration networks using the spatial lenses of countries would completely neglect the strongly unequal distribution within countries, and the spatial concentration in certain geographical areas within countries.
iii) At the same time, secondary data on R&D activities are often only available at country level, or at regional levels intersecting functional metropolitan areas (e.g. NUTS regions in Europe, see Lepori et al 2021).
iv) Therefore, we apply an innovative approach in this study put forward by a group of geographers as introduced by Villard et al (2019), or in a similar vein OECD (2012), Villard and Revollo (2015) or Maisonobe et al. (2018).
v) In essence, the urban areas used as spatial configuration in this study have been built based on several sources of information:

- 667 Functional Urban Areas in Europe (EU-28): built following the core analysis by density and with the addition of dependent areas (Lavalle, Kompil, & Aurambout, 2015);
- 17 Urban Areas for European associated countries: Urban Audit 2011-2014 (European Commission Eurostat, 2015);
- 104 Functional Urban Areas: from OECD, for Mexico, Colombia, South Korea, Japan, Chili, Canada, Australia (Brezzi, 2012; OECD, 2012);
- 450 Urban areas in US: US Census Bureau, Cartographic Boundary of Urban Areas, 2017 (United States Census Bureau – Geography Program, 2017);
- 375 Chinese Urban Areas: from Beijing City Lab, from an analysis of population density (based on BCL Data 19) in 2012 (Long & Shen, 2016);
- 2,587 Urban Areas for the rest of the world: Natural Earth, “derived from MODIS satellite data at 1 km resolution”, based on a method to capture dense light areas on earth (Patterson & Vaughn Kelso, 2014; Schneider, Friedl, & Woodcock, 2003).

vi) These about 4,200 urban areas have been combined in one spatial layer for a global perspective. Note that an urban area is defined as a spatial unit with more than 50,000 inhabitants.
vii) Then, rural areas have been added using a regional layer put forward by (Patterson & Vaughn Kelso, 2014) to fill portions of geographical space which are outside urban areas. Note that such rural areas can be very large when population is rather distributed in a number of cities below the threshold of 50,000 inhabitants (this explains the possibility that sometimes rural areas can also be among the top patent producers).
viii) Geocoding tools have then been mobilized to assign all patents to more the global urban and rural areas based on the inventor addresses given in the patents (using the Cortext tool, see https://www.cortext.net/).
ix) Finally, 41,397 inventor locations are assigned to 933 areas that locate at least one robotics patent inventor (i.e. the 900 is not a standard but simply the number of areas featuring robotics patent activity worldwide).
4. ***In note 4 (p.7), it is pointed out that ‘rural areas are included in the data, but patents are almost exclusively recorded to urban areas.’. However, in Tab.1, Aichi Rural is on the forth position. Does it reflect the inconsistency of the data sample?***

Also, in relation to the previous comment this comment stimulated clarify precisely the wording describing the spatial units under consideration and to explain the regional breakdowns much better in the revised manuscript.

i) As described in the previous response rural areas have been added using a regional layer put forward by (Patterson & Vaughn Kelso, 2014) to fill portions of geographical space which are outside urban areas.
ii) However, such rural areas can also have a very high population when it is composed of a number of cities below 50.000 inhabitants (as corresponding to the OECD definition, see OECD 2012)
iii) The notion of rural can then be bit misleading; for instance for Aichi, we have the situation that most inventors are located outside of the metropolitan centres Nagoya and Toyota, but this is still an urban environment, but labelled as rural due to the data assignment mechanics.
iv) To avoid this misunderstanding, we have erased the notion of “.rural” in the labelling of these areas and alternatively use the notion of ‘other’, to not ambiguously indicate to the reader that these are actually areas of rural character. The new region label indicates that the unit is referring to an area without subareas that are identified as coherent urban areas, e.g. ‘Other Bayern’ (before being labelled as Bayern.rural). ‘Other Bayern’ refers to Bayern without the urban area Munich, but Other Bayern is not only of rural character but also has a number of medium sized cities with R&D).
v) Still note more than 90% of all patents are recorded to urban areas by definition, so this is almost exclusively an urban phenomenon.
vi) Together with the changed labels, we have precisely clarified these issues by completely revising the description of the spatial configuration and make the breakdown clearer to the reader (see Section 3 and footnote 4).

**Reviewer #2**

1. ***Introduction***

***1.1-The growth of the robotics industry is presented as an opportunity for economic growth and competitiveness. However, there is no acknowledgement of the discussion about the societal challenges implied by it, particularly in terms of job losses (Frey and Osborne, 2017; Acemoglu and Restrepo, 2020), but more widely in the whole re-organization of societal functions and their impact in urban life (Macrorie et al., 2019). I understand that these discussions are not your key concern; however, you say that robots could address important societal challenges, but at the same time you might acknowledge that they could generate other problems.***

We agree with the referee that we completely missed this aspect. Based on this comment we reworked the characterisation of the robotics sector in Section 2 of the revised manuscript, adding some arguments on limitations and potential threats of robotics in order to put the discussion on economic and technological opportunities into perspective. Our answer is as follows.

i) *Robotics* are widely discussed, both in scientific literature as well as in the policy realm as one of the most crucial breakthrough technologies, i.e. bearing the potential for automation and therefore productivity increases across different manufacturing sectors, but also for addressing major societal challenges of our times (e.g. ageing, environmental threats, etc.).
ii) However, clearly robotics may have strong social consequences, in particular in labour markets and their transformation; for instance, labour shares may decline relatively in industries that are more amenable to automation (Acemoglu and Restrepo 2020), but at the same time new jobs will be created, though clearly requiring more complex and intellectual skills, and probably occurring in other geographical locations (Magnani 2022).
iii) We have added these arguments in the revised version of the manuscript that mostly was geared towards the economic and social opportunities before, neglecting these kinds of potentially negative social consequences pointed out by the referee.
iv) However, for the main essence of the study this is not a core argument, relying on the fact that, first, the importance of robotics industries has been increasing tremendously (see e.g. Shukla and Shukla 2012), and, second, that robotics are a highly R&D intensive segment and therefore may be subject to very specific geographical places of knowledge production, but also to increasing R&D collaboration, given the increased complexity and specialised competences needed for innovation (Goetze, 2010), also in robotics.
v) The changing geography of robotics R&D, both locations (hot spots) as well as global collaborations (networks), is largely unknown yet, at least to our knowledge in the systematic and geographically detailed way as put forward in the study at hand.

***1.2- The objectives of the study are somewhat general and not very connected to a gap in the literature. You say at page 3 that: “few studies exist that systematically trace the global landscape of robotic innovations and the related global R&D collaboration networks behind it”. Yet this does not explain why we need more studies on this, and what you would add to the existing literature. The fact that robotics are a very innovative technology is not sufficient, as other technologies could also be interesting to study including bio-engineering, artificial intelligence, internet of things or others. Also, many studies have geocoded patents, so I think you should play down the extent to which this is new, or explain how it differs from other contributions in this respect (see for example: Morrison et al., 2017; de Rassenfosse et al., 2019; Miguelez et al., 2019).***

We are grateful for this comment and the pointers made. The remarks stimulated us to revise and clarify the motivation but also the contribution of the study at hand more consistently, along the following main lines

i) The focus of the study at hand is on the geographical dynamics of global robotics R&D, identifying and characterising the changing landscape of R&D hotspots but also networks in robotics.
ii) In this respect, the study is unique being – at least to our knowledge – the only one looking at the geography of robotics and underlying networks at comparable detailed geographical scales and from a global perspective.
iii) Being considered as one of the most important breakthrough technologies of our times, providing fundamental insights into the changing geography of robotics R&D is a gap in the geography of innovation literature per se, also and even though this may be the case for other relevant technologies as well.
iv) In addressing this gap, we are combining a regional technological specialisation perspective with concepts from R&D collaboration networks, jointly characterising the global geographical dynamics of robotics hot spots and networks.
v) Though the study is explorative and descriptive in its essence, the results not only enrich the empirical literature in this field, but also bear some policy implications in terms of strategic R&D partnering or robotics R&D funding (e.g. from an EU perspective). At the same time, it paves the way for future research shifting attention to an explanation of the observed trend, for instance by econometric or network statistical modelling.
vi) We agree with the referee that there are studies putting forward geocoded patents at global levels (e.g. the ones mentioned by the referee), but few exploit these patents in the context we are putting forward with this study (both in terms of the sector addressed and the analytical network perspective on co-patents as marker for R&D collaboration between urban areas).
vii) We have tried to articulate the contribution more clearly in the revised version of the manuscript. In this context, we also take the pointer of the referee in his first sentence as main marker for the motivation and contribution in closing gaps in the literature, saying that the study “makes a timely and relevant contribution to make sense of the spatial emergence of this new industry that is already having profound impacts on the economy”.

***1.3- Audretsch and Feldmann, 1996: Reference is missing in bibliography***

Thanks, added!

2. ***Literature review***

***2.1- Related to the previous remark, the paper should be better embedded in the relevant literature. In fact, you discuss mainly applied contributions to the topic of robotics, but your motivation for the study is to understand the geographical dimension of R&D activities in this sector. You mention collaboration networks and knowledge exchange dynamics, and there is a huge literature on economic geography and the geography of innovation of which you could at least mention some of the main traits, authors and debates. I can’t resume here this literature but it includes discussions on the extent to which different forms of proximity favor networking, on knowledge complexity, on regional branching and relatedness and many other topics (you might check Balland and Boschma, 2021 for a recent application of some of these concepts on Industry 4.0 technologies, including robotics). These approaches are likely not all necessary for your point, but I believe you should ground your contribution onto some conceptualization of R&D and innovation dynamics in space, that is now lacking, and the related debates to which your paper contributes.***

We are grateful for this comment completely opening our eyes that we indeed missed this to a substantial extent in the original version of the manuscript (ironically even despite the author team has contributed to the literature the referee is referring to by themselves). We revised along the lines suggested by the referee, mainly in Section 2 but also at other places (introduction, conclusions):

i) We fully agree that the relation to theoretical debates and embedding in the literature on R&D collaboration networks has been developed just insufficiently in the original manuscript, also in conceptual terms motivating the usage of co-patents as relevant marker for R&D collaboration networks.
ii) We have substantially revised the manuscript, embedding much more comprehensively the study in theoretical literature and previous works.
iii) This is mainly done in a substantially extended Section 2, but also in the discussion and conclusions section.
iv) The main theoretical arguments – strongly driven by innovation economics and the literature on innovation systems – are assumptions stressing the increased networked character of R&D processes (see Powell and Grodal 2005, among others), on the one hand, and that such R&D collaborations may become increasingly internationalised in times of global innovation competition and chains (see Dachs et al. 2014),
v) Moreover, we stress arguments – as suggested by the reviewer – from the Economic Geography literature, emphasizing the role of spatial proximity and other spatial effects of for R&D collaboration networks (see Scherngell 2021 for an overview).
vi) Arguments raised there are actually also quite visible in this study, but have – as correctly observed by the reviewer – not been put into perspective in the first version of the manuscript.
vii) Actually the geographical logic is quite obvious looking at some of the results, e.g. the much stronger collaboration links within countries, but also a more intensive interaction within Asian countries than e.g. between Asia and Europe.
viii) Let us also note that we extend the theoretical debate also from a network science approach (see Newman 2001, among others), stressing that real-world social networks may increase in density and clustering over time due to specific network structural mechanisms.
ix) Related to this, we stress in the robotics context of the study that such networks may theoretically be of particular relevance in the rapidly changing robotics sector, as networks are often viewed as promising means to reduce uncertainty and risk in very dynamic environments (see Fischer 2003, among others).
x) Finally, we also use these selection of ideas to better highlight the limitations of the current study, more clearly articulating what it actually says, but what it does not (in delimitation to previous works, among them those mentioned by the referee), and in this context bringing in new future research perspectives (e.g. spatial interaction modelling in the geography context, exponential random graph modelling in the network science context).

***3.	Methods***

***3.1- Perhaps you could explain better the definition of urban areas that is used, or refer to an established definition (OECD?).***

We agree that there are some unclarities and inconsistencies in the description of the spatial configuration. We have clarified this issue in the methodology section of the revised manuscript (see Section 3). The main points in this context are the following:

i) On the one hand, when analysing the geography of R&D, a country perspective is too rough given the substantial regional disparities within countries. On the other hand, secondary data on R&D are usually, if at all, just available at the level of administrative regions, still often at a too rough level of granularity (e.g. provinces in China).
ii) Therefore, we apply an innovative approach in this study put forward by a group of geographers as introduced by Villard et al (2019), or in similar vein by OECD (2012), Laredo (2016), Villard and Revollo (2015) or Maisonobe et al. (2018).
iii) In essence, the urban areas used as spatial configuration in this study have been built based on several sources of information:

•	667 Functional Urban Areas in Europe (EU-28): built following the core analysis by density and with the addition of dependant areas (Lavalle, Kompil, & Aurambout, 2015);

•	17 Urban Areas for European associated countries: Urban Audit 2011-2014 (European Commission Eurostat, 2015);

•	104 Functional Urban Areas: from OECD, for Mexico, Colombia, South Korea, Japan, Chili, Canada, Australia (Brezzi, 2012; OECD, 2012);

•	450 Urban areas in US: US Census Bureau, Cartographic Boundary of Urban Areas, 2017 (United States Census Bureau – Geography Program, 2017);

•	375 Chinese Urban Areas: from Beijing City Lab, from an analysis of population density (based on BCL Data 19) in 2012 (Long & Shen, 2016);

•	2,587 Urban Areas for the rest of the world: Natural Earth, “derived from MODIS satellite data at 1 km resolution”, based on a method to capture dense light areas on earth (Patterson & Vaughn Kelso, 2014; Schneider, Friedl, & Woodcock, 2003).

iv) These about 4,200 urban areas have been combined in one spatial layer for a global perspective. Note that an urban area is defined as a spatial unit with more than 50,000 inhabitants.
v) Then, rural areas have been added using a regional layer put forward by (Patterson & Vaughn Kelso, 2014) to fill portions of geographical space which are outside urban areas. Note that such rural areas can be very large when population is rather distributed in a number of cities below the threshold of 50,000 inhabitants (this explains the possibility that sometime rural areas can also be among the top patent producers).
vi) Geocoding tools have then been mobilized to assign all patents to more the global urban and rural areas based on the inventor addresses given in the patents (using the Cortext tool, see <https://www.cortext.net>).

***3.2- In the RTA formula, the right part at the denominator you sum over i twice, whereas you should sum over i and k. Also, it might be clearer if you put it graphically this way:***

***(From Boschma et al., 2015).***

Thanks for spotting the index mistake, corrected! We also changed the graphical layout of the equation as suggested.

***3.3- Page 8 last paragraph, the following phrase has some syntactic problem (missing verbs?): In general, SNA has come into fairly wide use for the analysis of social systems, been to be interpreted at the individual level of socially interacting individuals***

Thanks for spotting the mistake, corrected!

***3.4- At page 9, first paragraph, adjacency matrix as defined by equation (1 2)***

Thanks for spotting the mistake, corrected!

***3.5- At pages 9 and 10, the lists of network measures is a bit too long and probably not necessary as they are fairly well known. You could refer to Wasserman and Faust for a full definition and just list the measures used and what they show in general, using half the space***

We agree and reworked the part making the text more stringent (reduced the text and partly put in footnotes, overall by about 50% as suggested by the referred). Note however that this is always a bit of a trade-off between readers (and reviewers) where the readers tend preferring to have these basics in the main text instead of searching these mechanics up in the literature. Those readers not interested simply skip it.

***4.	Results***

***4.1- Table 1 is unclear. In fact, while the title is “RTA analysis”, the first three fields are not RTA indexes, because RTA appears only in the last field. This raises two questions: if the first three fields represent patent counts, why are they not integer numbers? If you have used fractional counts, assigning a proportional contribution to inventors in different cities, you should mention it before, so the reader can interpret these results. Second question: how can you say anything about the evolution of specialization if you only show RTA for the period 2012-2016 and not the previous one? The increase in absolute patent numbers could be simply an indication of the aggregate growth of patenting activity.***

Thanks for pointing out these inconsistencies that really motivated us to describe better (empirically and in the text) the evolution patterns. We have corrected and clarified as follows:

i) We have extended the header of Table 1 to “R&D hot spots in robotics (top ten) and their relative specialisation (RTA)”
ii) Indeed, we use fractional counting of patents, rather than full counting, and agree that this is not sufficiently described in the text. We have clarified this in the text and also directly in the notes to Table 1. Note that we prefer fractional counting as the full counting method would strongly over-estimate R&D in the areas given the high number of inventors per patent. With the fractional counting, we go for the inventor share, i.e. we count the fraction of inventors located in one area to reproduce the actual number of patents and, by this, not overestimate R&D activity.
iii) In the description of the trends, we do not speak about evolution of the RTA, but just of patent counts. However, we agree that this is an interesting element and have added the RTA of the previous periods to Table 1.
iv) Moreover, we have put the aggregate patent growth in robotics into perspective against the total patent growth of the areas.

***4.2- Related to this, I have some doubts that these results can support the findings you report at page 11. Particularly, in finding number 2 you say that two areas have shifted their focus to robotic R&D, but even though these numbers could suggest it, they are not enough to state it confidently. These data only say part of the story, so that for example, this might be part of a common growth in robotic specialization, or other technology fields in these urban areas might be growing even more than robotics. Hence, I suggest to nuance a bit more these statements.***

Thanks for pointing out these critical issues, and this is strongly related to the previous remark. We have mainly addressed this by putting the results into perspective, in particular in terms of what are actually assumptions that may be verified in further research, but also by adding the empirical results as described in the response to the previous remark.

i) In the finding commented by the referee, we hypothesize that the areas Stockholm and Kitakyushu shifted their focus to robotic R&D activities and expended their specialization advantage considerably more recently.
ii) We have underlined this result by adding the RTA values for the previous period (see also the response to the previous remark), where we can find the RTA for these two areas increases immensely between the observed time periods (for Stockholm the RTA is more than doubling between the periods. Kitakyushu has even increased RTA by a factor of 13.

***4.3- Page 12, first paragraph: you say that “most urban areas show a strong specialization in the automotive industry”, but what you want to say is that applicants in these regions are mostly automotive companies. Your data do not allow you to say much about the overall specialization of these areas.***

Agree also put into perspective!

***4.4-Same page-same paragraph as above, why would you expect universities or research organization to be at the top of the list? Also, data about higher share of research facilities in the Asian cities you mention is not disclosed.***

These points we have been clarified in the revised manuscript. Actually, this mainly creeped out from some sloppy textual description in the original version of the manuscript, and some missing empirical elements:

i) We do not expect universities and/or public research organisations to be among the top patent applicants which is clearly underlined by the overall empirical results. The sentence “it is most striking that research facilities (i.e. universities or public research organizations) are not very active …” may have been misleading in that respect, and we have re-formulated it to “As expected from previous works and underlying rationales in R&D processes, public research organisations are not very active in…”.
ii) At the same time, we find an interesting element that for some Asian countries this seems not to be the case.
iii) We agree that we should be more precise here and underline this with some empirical illustrations. In this context, we have replaced Asian countries by specifying China and South Korea and added the top public patent holders in footnote 11.
iv) We think this is an interesting pattern and outcome of the empirical analysis, pointing to a more early stage phase in the evolutionary path of these countries as compared to ‘traditional’ robotics countries like the US, Germany or Japan, and in this earlier stages public R&D is more prevalent. In addition, this may partly be a strategic issue, to keep patents in public research organisations, e.g. for the Chinese case.

***4.5- The table A5 you mention at page 11 does not exist in the appendix. Is it A3?***

Exactly, corrected!

***4.6- Figure 1 and the related commentaries suggest that there has been a general increase more than decrease of specialization in robotics (related to commentary 4.2). This should be acknowledged somewhere.***

We agree with the referee that this is a very interesting point, but somewhat underrepresented in the discussion since it actually is one of the main messages of the study.

i) Actually, our empirical analysis is the first to demonstrate at a very detailed level that not only robotics R&D is growing strongly in absolute and relative terms (see also our comparison to total growth and other sectors, response to comment 6.1).

ii) Looking at the RTA results, it can be seen that the average rank change is 12.08, indicating that there are on average more urban ascending areas than descending ones.
iii) This dynamic is highly interesting, because the shift is by far not equally distributed in geographical space. So, the average RTA increases, while at the same time this average increase is subject to new emerging areas, in particular in China, that have not been specialised in the earlier years of the observed time period.
iv) This space-time shift in robotics R&D is one of the main results of the study at hand, and we have made this much more prominent – stimulated by the remark of the referee – in the revised version of the manuscript, both in the abstract, in the results discussion itself and the conclusions.
v) However, at the same time we can only speculate about the main driving forces behind these spatial dynamics and therefore point in the last paragraph on future research to the demand for an explanatory approach identifying statistically these drivers.

***5.	Network results***

***5.1- In figure 2, in the notes it is not clear to what “number” link size refers to.***

Thanks for the pointer, we have corrected this (and also removed some none interpretable graphical values coming from the visualisation software). Simply node size corresponds with an area´s degree, thickness of lines to the interaction intensity between two areas.

***5.2- There doesn’t appear to be any table A8 or figure 5.***

We have removed the reference A8. “Figure 5” was a wrong reference; we have corrected it to “Figure 2”.

***5.3- Page 17, third paragraph: “places on earth” sounds vague.***

Agree, we have reframed the whole sentence, referring to geographical dynamics observed for global R&D collaboration networks including the changing role of several urban areas worldwide, characterized both by ascending and descending ones in terms of network centrality.

***5.4- In this section you use three different measures of centrality. To which in particular do you refer when you talk of “network centrality” (p.15)? Do you consider them as being equally important or you give priority to one over others? What is the value-added of considering different measures of centrality?***

This is an important remark that motivated us to clarify the interpretation in this respect. Actually, these different centrality measures characterize different qualities of embedding in a network.

i) Local network measures are mobilized to capture the role and embedding of individual nodes in networks. While centrality measures are usually directly related to the intensity a node is networked in terms of its quantity of links, different definitions of centrality are able to capture different qualities of these inter-linkings (see e.g. Hu et al. 2013).
ii) In the study at hand, we follow previous research on R&D collaboration networks (see e.g Hu et al. 2013, Wanzenböck et al. 2015, Scherngell et al. 2021) and look at the local interaction intensity of a node (urban area) in the network by means of degree centrality, the global embedding in terms of the connection of a node to other central nodes (Eigenvector centrality), and the ability to ‘bridge’ interaction between other nodes in terms of betweenness centrality.
iii) While clearly at an aggregated level of spatial entities, these measures of centrality are much more correlated as it would be the case when using individual organisations (or even research teams or researchers); but we still find some interesting differences between the three measures in the empirical setting of this study.
iv) For instance, it is shown that the more established areas (e.g. German ones) tend to have a higher Eigenvector centrality, i.e. they are more connected to a long established core than emerging areas, but also betweenness centrality (e.g. US and Japanese ones), i.e. they are able to act as bridges between other urban areas.
v) In contrast, the emerging urban areas (and here in particular Beijing, Guangzhou and Shanghai) appear in the top in more local embeddings (degree centrality), while still somewhat behind the top players in the other quality dimensions.
vi) Stimulated by the remark of the reviewer, we have seen that we have not sufficiently elaborated this discussion in the original version of the manuscript and have added these ideas, both in the results and the conclusions section, respectively.

***6. Discussion***

***6.1- Page 18 last paragraph, you say that robotics “has grown immense”. However, you should contextualize this finding, because we know that patenting in general has greatly increased in past decades. At the same time, growth in robotics could be relativized against growth in other emergent sectors. Otherwise, this claim does not appear very substantiated.***

This is a very good idea, and we follow the referee in that respect. We have underlined our claims with empirics, not only in the conclusions but also in the description of the robotics focus and the results section. The main points are the following:

i) We can observe a clear growth pattern for robotics patents. Patenting in absolute numbers has increased from 3,262 to 8,531 between the first and last period observed in this study. This equals a growth of 162% in absolute terms.
ii) Looking at the share of robotic on total patents (to account for the overall increase in patenting), we can find that this share increased from 0.13% in the first reporting period to 0.33% in the last reporting period by 148%.
iii) This growth in the share on total patents is also remarkable compared to other emergent sectors, e.g. as compared to all ICT (+15%), digital communication technologies (+64%), semiconductors (+7%) or the also the very fast growing technologies related to artificial intelligence (+121%). Other emerging technologies including Nanotechnology (-58%) or Biotechnology (-30%) even experienced a declining share on total patents.
iv) We have added this, not only in the discussion part but also in the characterisation of the robotics sector as a whole (also including the information about growth in economic demand and value added).

***6.2 –I do not see how the limited evidence presented in table A3 can support such vast claim as the fourth one. The remark is interesting, but you should support it with further evidence, perhaps including a larger number of patent applicants and extending research beyond the top 10 RTA areas.***

We thank the referee for the pointer and have revised the manuscript accordingly. On the one hand, we have put the statements into perspective, on the other hand, we have added some empirical evidence on it by looking at CPC subclasses emerging urban areas are more active in as compared to established urban areas.

i) We agree that without further empirical evidence the statement is speculative, and our formulation was too apodictic in that respect. We have accordingly nuanced to text clearly articulating it as assumptive, but a meaningful one coming out of the empirical results.
ii) Moreover, we looked into some further empirics to underline these conclusions, by comparing counts for CPC subclasses (as listed in Table A1) between the established regions under the Top 10 (i.e. those that have already been on top in the first period) to the emerging ones (those that appeared in the Top 10 just in the last period).
iii) The results show that indeed the emerging urban areas tend to patent in other CPC robotics classes (mainly B25 classes) than the established ones who patent mostly in classical automotive ones (in particular B60).
iv) Looking at the importance of CPC subclasses as a whole, this can be supported going beyond the Top 10 areas. We find that the B25 increase their share in total robotics patenting from 10% (2002-2007) to 15% (2012-2016).
v) Since this small empirical analysis does not fit in the discussion, but just underlines our fourth conclusion, we have put these results in the R&D hot spots analysis as additional marker (see Section 4).

***6.3- Data in support of the fifth claim is not presented (see comment 4.4).***

Agree, see our response to comment 4.4! We have added these empirics underlining the important role of public research organisations in Chinese robotics R&D to the text.

***6.4- The discussion does not explain how research findings relate to existing debates and research directions in the literature (see also comments 1.2 and 2.1).***

As also stressed in our response to comments 1.2 and 2.1, we fully agree and have added relations to existing literature, in particular on the geography of R&D collaboration networks, also in the discussion section (e.g. in terms of the growth of R&D internationalisation that we confirm for robotics in the study hand, the geographical logic inherent in such networks, the network configurational mechanics, or the spatial dynamics and shifts in global R&D). This is also put into perspective against future research perspectives, where we stress as one of the main points to follow-up with an explanatory framework shifting attention to a more profound explanation rather than description of the observed drivers, e.g. in a spatial interaction or network modelling context.

## References

Acemoglu, D., & Restrepo, P. (2020). Robots and jobs: Evidence from US labor markets. Journal of Political Economy, 128(6), 2188-2244.

Audretsch, D. B., & Feldman, M. P. (1996). R&D Spillovers and the Geography of Innovation and Production. The American Economic Review, 86(3), 630–640. http://www.jstor.org/stable/2118216

Brezzi, M. (2012). Redefining urban: a new way to measure metropolitan areas. OECD Publishing. Retrieved from http://www.oecdbookshop.org/browse.asp?pid=title-detail&lang=en&ds=&ISB=9789264174054

Dachs, B., Stehrer, R., & Zahradnik, G. (2014). The internationalisation of business R&D. Edward Elgar Publishing

European Commission Eurostat. (2015). Urban Audit 2011–2014. Retrieved from https://ec.europa.eu/eurostat/web/gisco/geodata/reference-data/administrative-units-statistical-units/urban-audit#ua11-14

Fischer, M. M. (2003): The new economy and networking, Jones, D. C. (ed) New Economy Handbook, San Diego CA, Academic Press: 343-367

Goetze, C. (2010). An empirical enquiry into co-patent networks and their stars: The case of cardiac pacemaker technology. Technovation, 30(7-8), pp. 436-446. doi:10.1016/j.technovation.2010.03.003

Hoekman, J., K. Frenken and F. Van Oort (2009). "The geography of collaborative knowledge production in Europe." The Annals of Regional Science **43**(3): 721-738.

Hu, Y. (2006). Efficient and high quality force-directed graph drawing. Mathematica Journal. 10. 37-71

Hu, Y., Scherngell, T., Man, S. N., & Wang, Y. (2013). Is the United States still dominant in the global pharmaceutical innovation network?. PLoS One, 8(11).

Hussler, C., & Rondé, P. (2007). The impact of cognitive communities on the diffusion of academic knowledge: Evidence from the networks of inventors of a French university. *Research Policy*, *36*(2), 288-302.

Keisner, C., Raffo, J., & Wunsch-Vincent, S. (2015). Breakthrough technologies – Robotics, innovation and intellectual property. World Intellectual Property Organization (WIPO). Retrieved from https://www.wipo.int/edocs/pubdocs/en/wipo_pub_econstat_wp_30.pdf

Lata, R., T. Scherngell and T. Brenner (2015). "Integration Processes in European Research and Development: A Comparative Spatial Interaction Approach Using Project Based Research and Development Networks, Co‐Patent Networks and Co‐Publication Networks." Geographical Analysis **47**(4): 349-375.

Lavalle, C., Kompil, M., & Aurambout, J.-P. (2015). UI – Boundaries for the functional urban areas (LUISA Platform REF2014). Retrieved from http://data.europa.eu/89h/jrc-luisa-ui-boundaries-fua

Lepori, B., Laredo, P., Scherngell, T., Maynard, D., & Guerini, M. (2019). The spatial distribution of knowledge production in Europe. Evidence from KET and SGC. In 17th International Conference on Scientometrics and Informetrics, ISSI 2019 (Vol. 2, pp. 685-690).

Long, Y., & Shen, Y. (2016). Mapping parcel-level urban areas for a large geographical area. Annals of the American Association of Geographers, 106(1), 96–113. Retrieved from http://arxiv.org/abs/1403.5864%0Ahttp://www.arxiv.org/pdf/1403.5864.pdf

Magnani, M. (2022). The Technological Revolution: Professions at Risk and New Jobs. In Making the Global Economy Work for Everyone (pp. 53-71). Palgrave Macmillan, Cham.

Maisonobe, M., Jégou, L., & Eckert, D. (2018). Delineating urban agglomerations across the world: a dataset for studying the spatial distribution of academic research at city level. Cybergeo. https://doi.org/10.4000/cybergeo.29637

Newman, M. E. (2001). Scientific collaboration networks. II. Shortest paths, weighted networks, and centrality. *Physical review E*, *64*(1), 016132.

OECD (2007): Science, Technology and Industry Scoreboard 2007, (<https://doi.org/10.1787/sti_scoreboard-2007-en>)

OECD. (2012). Redefining “Urban”: A New Way to Measure Metropolitan Areas. OECD Publishing, (September), 1–9. https://doi.org/10.1787/9789264174108-en

OECD (2017): Science, Technology and Industry Scoreboard 2017, (<https://doi.org/10.1787/9789264268821-en>)

Patterson, T., & Vaughn Kelso, N. (2014). Natural Earth, 1–2. Retrieved from https://www.naturalearthdata.com/about/terms-of-use/

Powell, W. W., & Grodal, S. (2005). Networks of innovators. The Oxford handbook of innovation, 78

Scherngell, T. (2021). The geography of R&D collaboration networks. Handbook of Regional Science. M. M. Fischer and P. Nijkamp. Berlin, Springer.

Scherngell, T. (ed.) (2013): The Geography of Networks and R&D Collaborations. Springer-Physica Verlag, Berlin-Heidelberg-New York

Schneider, A., Friedl, M., & Woodcock, C. E. (2003). Mapping Urban Areas by Fusing Multiple Sources of Coarse Resolution Remotely Sensed Data. Photogrammetric Engineering and Remote Sensing Remote Sensing, 69(C), 1377–1386.

Shukla, M., & Shukla, A. N. (2012). Growth of robotics industry early in 21st century. International Journal of Computational Engineering Research, 2(5), 1554-1558

UKIPO. (2014). Eight Great Technologies - Robotics and Autonomous Systems: A Patent Overview. London: UK Intellectual Property Office

United States Census Bureau – Geography Program. (2017). Urban areas. Retrieved August 22, 2019, from https://www.census.gov/programs-surveys/geography/geographies/mapping-files.2017.html

Villard, Lionel, Opsina, Juan Pablo & Medina, Luis Daniel (2019). CorText Geospatial Exploration Tool. ESIEE Paris, Paris Est University. <https://docs.cortext.net/cortext-geospatial-exploration-tool/>

Villard, L., & Revollo, M. (2015). GeoClust: a tool to study the geographical aggregation of activities. CorText. Retrieved from https://www.cortext.net/geoclust-a-tool-to-study-the-geographical-aggregation-of-activities/

Wanzenboeck, I., Scherngell, T., & Brenner, T. (2014). Embeddedness of regions in European knowledge networks: a comparative analysis of inter-regional R&D collaborations, co-patents and co-publications. *The Annals of Regional Science*, *53*(2), 337-368.
