# Supplementary material for: The geographical dynamics of global R&D collaboration networks in robotics: Evidence from co-patenting activities across urban areas worldwide
Source: PLoS One. 2023 Apr 13;18(4):e0281353. doi: 10.1371/journal.pone.0281353 (PMC10101514; doi:10.1371/journal.pone.0281353)
Supplement: Attachment — Submitted filename: Paper_review2.docx [file pone.0281353.s004.docx]

Thank you for submitting this revised version of your paper. Your modifications have improved the paper substantially, and mostly address my previous remarks. However, I still think you need to make an extra effort to anchor your contribution better onto existing literature. Your empirical results are very valuable, and I believe you can connect them to existing theoretical debates, but the link needs to be made more explicit (see remarks 1.2, 2.1, 6.3). Apart from these remarks, the rest are secondary corrections.

**1. Introduction**

1.1- When you discuss mission-oriented research policies you may want to mention that robotics is part of the Key Enabling Technologies identified by the EU (KETs), and the related definition of General Purpose Technologies (GPT). See Montresor and Quatraro (2017).

1.2- Research objectives are still not connected to a literature debate. I see that you motivated better the fact that robotics is an important industry that has impact on the economy, which makes your paper timely and societally relevant. Yet I still feel as your main goal is describing the robotics industry per se, and not as a case study to investigate geographical specialization or network dynamics. You can have purely empirical objectives, but I am convinced that the paper would benefit if you could make explicit also a theoretical one.

**2. Literature review**

2.1- Thank you for integrating literature on the societal impact of robotization, and for adding references to debates in economic geography and network science. However, the reader still does not understand how you integrate a geographical and “a-spatial” perspectives, and what you add to existing debates on these topics (e.g. Glückler, 2007; ter Wal and Boschma, 2011). You don’t seem to use the literature you presented to support a specific point beyond the fact that robotics is a strategic industry whose spatial configuration has not been studied.

2.2- This section is rather long, so you might benefit from creating thematic sub-sections of this chapter, to make the argument clearer.

**3. Data and methods**

3.1- Thank you for clarifying the spatial delimitation and the issue of fractional counts. It’s much clearer now.

3.2- Just before the RTA equation, you write: “Regional specialization in robotics exists when a spatial uses”. You probably forgot something.

**4. Results**

4.1- Tables are much clearer with the contextualized figures.

4.2- In figure 1, the line looks as a regression line, but not one that identifies, as you say, “movements of ascending (above the line) and descending (below the line) urban areas” in terms of RTA. If you wanted to show that, the line should be the diagonal, with slope coefficient of 1 and intercept 0. You can easily check this by manually considering the points in your figure. For example, the one I highlighted here below should be on a descending trend according to you: instead, its rank is around 150 in period 07-11 and around 130 in 12-16, so rank is ascending. The line I plotted, by contrast, rightly identifies the point as being ascending. Actually, this shows that an even higher share of cities is increasing their specialization in robotics, with respect to your previous line. It also shows that the top cities perfectly align with the line, so that the hierarchy at the top is rather stable.

**5. Network dynamics**

5.1- The discussion of different centrality measures is very pertinent and insightful, thank you.

5.2- Thank you for clarifying the legend of network images. However, it’s not clear to which interactions correspond the thickness of lines. You cite 5 pages earlier that it’s co-patenting intensity, but a reminder about that also in the graph’s legend would make it clearer.

5.3- Paragraph 4, the last sentence: is it an argument of local buzz vs global pipelines or rather one of gatekeeping? I think it’s more about cities being national gatekeepers with respect to global flows.

5.4- Paragraph 6, the ability to “bridge” instead of “bride”.

**6. Discussion and conclusions**

6.1- In this section, I suggest you separate more clearly the empirical results of your paper from the conceptual and policy contributions. Right now, you mix empirical results (paragraphs 3 and 5) with conceptual ones (paragraph 4) and policy (paragraph 6). I think you should organize it better by providing empirical results first, then conceptual/policy results.

6.2- Furthermore, I think you should add a brief discussion of the limitations of your study and link it more coherently to a discussion on research perspectives, that right now is a bit lost into policy conclusions.

6.3- In relation to your contribution to the literature, paragraph 4 does not answer to the question: what is the new contribution of this study to the relevant literature? You address some points, but the discussion is still too vague. For example: “a certain logic in terms of proximity dimension”. Which logic do you refer to? Also, the “specific network structural mechanisms” do not look so specific but rather general (growing integration/changing roles of nodes). I understand that your study is descriptive, and that your results partly confirm existing studies, which is fine. However, you could point to the conceptual advances that you provide besides the empirical novelty of the case study. I think that findings on changes in specialization and different forms of centrality are interesting to the debate on regional diversification and path-dependence (Grillitsch et al., 2018; MacKinnon et al., 2019). This relates to the capability of innovative cities to retain their edge or lose it because others are better at acquiring new technological innovations. In fact, you show that German cities are very central in terms of Eigenvector centrality but, for example Stuttgart, loses betweenness centrality. This is an interesting empirical finding that you could link to the debates that are at the core of contemporary economic geography such as the capability of cities and regions to branch into new technologies, (smartly) diversify and renew their productive structure. You could also expand a bit more on insight number four (automotive vs other robotics sector) which could also be a relevant point to path-dependence debates.

**References**

Gluckler, J., 2007. Economic geography and the evolution of networks. Journal of Economic Geography 7, 619–634. <https://doi.org/10.1093/jeg/lbm023>

Grillitsch, M., Asheim, B., Trippl, M., 2018. Unrelated knowledge combinations: the unexplored potential for regional industrial path development. Cambridge Journal of Regions, Economy and Society 11, 257–274. <https://doi.org/10.1093/cjres/rsy012>

MacKinnon, D., Dawley, S., Pike, A., Cumbers, A., 2019. Rethinking Path Creation: A Geographical Political Economy Approach. Economic Geography 95, 113–135. <https://doi.org/10.1080/00130095.2018.1498294>

Montresor, S., Quatraro, F., 2017. Regional Branching and Key Enabling Technologies: Evidence from European Patent Data. Economic Geography 93, 367–396. <https://doi.org/10.1080/00130095.2017.1326810>

Ter Wal, A.L.J., Boschma, R., 2011. Co-evolution of Firms, Industries and Networks in Space. Regional Studies 45, 919–933. <https://doi.org/10.1080/00343400802662658>
