# Supplementary material for: The geographical dynamics of global R&D collaboration networks in robotics: Evidence from co-patenting activities across urban areas worldwide
Source: PLoS One. 2023 Apr 13;18(4):e0281353. doi: 10.1371/journal.pone.0281353 (PMC10101514; doi:10.1371/journal.pone.0281353)
Supplement: Attachment — Submitted filename: Response_R2.docx [file pone.0281353.s005.docx]

**Response to reviewer comments (Manuscript ID PONE-D-21-39533R1)**

We thank the referees for acknowledging that the revised manuscript has improved based using their valuable comments. We are grateful for the very detailed remaining remarks by one referee that are highly appreciated. In what follows we shortly reflect on how we dealt with the remaining comments. On a general note, we agree with all comments raised and have more or less directly incorporated these very detailed and very structured recommendations. The remarks do not only make the work more solid, but also more attractive by adding missing or not sufficiently pronounced links to current debates in the literature the referee is rightly pointing to.

***1. Introduction***

*1.1- When you discuss mission-oriented research policies you may want to mention that robotics is part of the Key Enabling Technologies identified by the EU (KETs), and the related definition of General Purpose Technologies (GPT). See Montresor and Quatraro (2017).*

Thanks! We have added this pointer including the reference in the introduction in the passage where we describe that robotics has been increasingly addressed not only by the scientific, but also by the policy realm.

*1.2- Research objectives are still not connected to a literature debate. I see that you motivated better the fact that robotics is an important industry that has impact on the economy, which makes your paper timely and societally relevant. Yet I still feel as your main goal is describing the robotics industry per se, and not as a case study to investigate geographical specialization or network dynamics. You can have purely empirical objectives, but I am convinced that the paper would benefit if you could make explicit also a theoretical one.*

We thank the referee for this remark that we have accounted for in the derivation of the objective for the revised manuscript. Though we keep the main objective as it is (observe and systematically describe dynamics of R&D collaboration networks in robotics at global scale with detailed geographical breakdown), we make now explicit that, with this new empirical insights, we aim not only to inform scholars empirically investigating R&D networks, and the policy community dealing with robotics as important field, but also enrich theoretical debates in the context as suggested by the referee, positioning this work as an insightful case illustrating spatial shifts coming with new specializations in R&D and related collaboration networks.

***2. Literature review***

*2.1- Thank you for integrating literature on the societal impact of robotization, and for adding references to debates in economic geography and network science. However, the reader still does not understand how you integrate a geographical and “a-spatial” perspectives, and what you add to existing debates on these topics (e.g. Glückler, 2007; ter Wal and Boschma, 2011). You don’t seem to use the literature you presented to support a specific point beyond the fact that robotics is a strategic industry whose spatial configuration has not been studied.*

We agree that the discussion about integrating ideas from geography and network science stays to much unrelated to the empirical analysis. We have tried to make clearer on how these two perspectives relate to our empirical work, saying that we lie in the tradition of the literature stream jointly investigating spatial configurations in relation to network structural mechanisms (see Neuländtner and Scherngell 2020 for a recent example in an explanatory context), but more from a descriptive perspective (for instance, by looking at spatial shifts of R&D hot spots, and how such spatial shifts in R&D specialization co-occur with network structural changes, e.g. centralities of regions in the network).

*2.2- This section is rather long, so you might benefit from creating thematic sub-sections of this chapter, to make the argument clearer.*

We agree indeed. This was resulting from the substantial first revision connecting the work to the different debates in the literature where we wanted to be sure to address the comments raised for the first revision in a sufficient manner. We have now added more structure to the subsection by introducing subtitles and making the text more stringent.

***3. Data and methods***

*3.1- Thank you for clarifying the spatial delimitation and the issue of fractional counts. It’s much clearer now.*

Thanks!

*3.2- Just before the RTA equation, you write: “Regional specialization in robotics exists when a spatial uses”. You probably forgot something.*

Thanks for spotting the issue! We have added “unit” after “spatial”.

***4. Results***

*4.1- Tables are much clearer with the contextualized figures.*

Thanks!

*4.2- In figure 1, the line looks as a regression line, but not one that identifies, as you say, “movements of ascending (above the line) and descending (below the line) urban areas” in terms of RTA. If you wanted to show that, the line should be the diagonal, with slope coefficient of 1 and intercept 0. You can easily check this by manually considering the points in your figure. For example, the one I highlighted here below should be on a descending trend according to you: instead, its rank is around 150 in period 07-11 and around 130 in 12-16, so rank is ascending. The line I plotted, by contrast, rightly identifies the point as being ascending. Actually, this shows that an even higher share of cities is increasing their specialization in robotics, with respect to your previous line. It also shows that the top cities perfectly align with the line, so that the hierarchy at the top is rather stable.*

We are extremely grateful for spotting this issue! It was indeed a regression line that has been added incorrectly. We have corrected the figure accordingly (and as correctly suggested by the referee). Actually, the corrected illustration even more underlines our argument (namely a higher share of cities is increasing their specialization in robotics) with a stable hierarchy on the top. The inferences on what concerns main ascenders and descenders did not change.

***5. Network dynamics***

*5.1- The discussion of different centrality measures is very pertinent and insightful, thank you.*

Thanks!

*5.2- Thank you for clarifying the legend of network images. However, it’s not clear to which interactions correspond the thickness of lines. You cite 5 pages earlier that it’s co-patenting intensity, but a reminder about that also in the graph’s legend would make it clearer.*

Thanks for the pointer. We have clarified directly under the graph and in the description of the figure the meaning of the lines (*co-patenting intensity*).

*5.3- Paragraph 4, the last sentence: is it an argument of local buzz vs global pipelines or rather one of gatekeeping? I think it’s more about cities being national gatekeepers with respect to global flows.*

Thanks for the pointer! Actually, it is a wonderful example of a local buzz vs. global pipelines structure. We have added the argument and refer, following the suggestion of the referee, to national gatekeeper cities.

*5.4- Paragraph 6, the ability to “bridge” instead of “bride”.*

Thanks, the typo has been corrected.

***6. Discussion and conclusions***

*6.1- In this section, I suggest you separate more clearly the empirical results of your paper from the conceptual and policy contributions. Right now, you mix empirical results (paragraphs 3 and 5) with conceptual ones (paragraph 4) and policy (paragraph 6). I think you should organize it better by providing empirical results first, then conceptual/policy results.*

We follow the referees’ suggestion and bring more structure to the discussion along the lines as suggested. We have in the revised manuscript put the empirical results in the front, condensing the main insights to one paragraph, while we shift attention to conceptual and policy related conclusions in the two paragraphs that follows. Overall, we have slightly shortened the final section and increased stringency.

*6.2- Furthermore, I think you should add a brief discussion of the limitations of your study and link it more coherently to a discussion on research perspectives, that right now is a bit lost into policy conclusions.*

We agree and have added respective limitations. As a core issue we raise that the study cannot derive but just speculate about drivers for the observed network dynamics. This paves at the same time the way for a future research program that needs to shift from an explorative to an explanatory framework, e.g. by introducing a spatial interaction or (temporal) exponential graph modelling perspective.

*6.3- In relation to your contribution to the literature, paragraph 4 does not answer to the question: what is the new contribution of this study to the relevant literature? You address some points, but the discussion is still too vague. For example: “a certain logic in terms of proximity dimension”. Which logic do you refer to? Also, the “specific network structural mechanisms” do not look so specific but rather general (growing integration/changing roles of nodes). I understand that your study is descriptive, and that your results partly confirm existing studies, which is fine. However**, you could point to the conceptual advances that you provide besides the empirical novelty of the case study. I think that findings on changes in specialization and different forms of centrality are interesting to the debate on regional diversification and path-dependence (Grillitsch et al., 2018; MacKinnon et al., 2019). This relates to the capability of innovative cities to retain their edge or lose it because others are better at acquiring new technological innovations. In fact, you show that German cities are very central in terms of Eigenvector centrality but, for example Stuttgart, loses betweenness centrality. This is an interesting empirical finding that you could link to the debates that are at the core of contemporary economic geography such as the capability of cities and regions to branch into new technologies, (smartly) diversify and renew their productive structure. You could also expand a bit more on insight number four (automotive vs other robotics sector) which could also be a relevant point to path-dependence debates.*

We are extremely grateful for these valuable remarks that not only enrich the conclusion section, but also the section containing the empirical results (where we have put some of the more detailed pointers of the referee). We are, on the one hand, making the too vague parts more concrete and graspable (e.g. by geographical logic we simply referred to the fact that spatial proximity seems to play – as expected – a very important role in network evolution, also in the robotics sectors). On the other hand, we connect this to the discussion of regional technological diversification and branching, where the study at hand could serve as an illustrative case to underline such mechanisms. In relation to the previous comment, we add this to the future research program, namely investigating how far the observed dynamics for specific regions are related to underlying diversification or more upscaling mechanisms.
